# Beyond Food Safety: Taxonomization of Private Initiatives to Design Healthier Supermarket Environments

**DOI:** 10.1007/s13668-025-00660-1

**Published:** 2025-05-28

**Authors:** Ana Ines Estevez Magnasco, Dominic Lemken, Hanna Ihli

**Affiliations:** 1 Group of Socioeconomics of Sustainable Nutrition, Institute for Food and Resource Economics, University of Bonn, 53111 Bonn, Germany; 2 Economic and Agricultural Policy, Institute for Food and Resource Economics, University of Bonn, Bonn, Germany

**Keywords:** Supermarket, Sustainable nutrition, Private policies, Taxonomny

## Abstract

**Purpose of Review:**

While policies targeting education in schools, marketing campaigns, and taxation strategies are of great importance in tackling our population’s malnutrition, there is growing concern about enhancing the supermarket environment to promote healthier food consumption. Supermarkets play a significant role in presenting food options. As gatekeepers of the food system’s impact on consumers’ health, they have the power to help people make better food choices. Recent shifts in the policy agendas in countries like Germany reflect this trend, with new nutrition-focused behavioural policies being legislated and implemented. However, there remains a lack of specific guidelines on how supermarket environments can be structured to promote healthier purchases. What strategies could enhance consumer well-being when making food choices?. Following the PRISMA framework, we harvested sources published since the SDGs (2015) release related to sustainable nutrition policies set by supermarkets. Using the INFORMAS modular structure framework, WHO action areas as guidance, and the reviewed documents, we created a taxonomy for policy initiatives that could improve the supermarket environment and promote healthy choices. Consequently, we studied the private policies of major supermarket groups (Rewe, Lidl, Aldi Nord, Aldi Sud and Edeka), examining their projects and campaigns designed to improve supermarket environments and encourage consumers to choose healthy food options.

**Recent Findings:**

From the taxonomy, comprised of the categories of campaigns, food reformulation, labelling, and marketing, the main categories of campaigns and labelling were thoroughly developed in all the analysed policies whereas food reformulation and marketing categories were analysed in 80% of them. Our analysis revealed that the implementation of sustainable nutrition policies appears to have been embraced by all supermarket groups providing general healthy and sustainable nutrition strategies, except for the Edeka. Notably, supermarkets have actively participated in the creation of private sustainable nutrition policies, presenting similar strategies across the board.

**Summary:**

However, while supermarkets have demonstrated commitment to promoting healthy and sustainable purchases, there is a need for greater coherence and alignment in reporting mechanisms to allow accurate comparison between initiatives. There´s an urgent need to focus on human welfare, where the purchase environment is healthy and promotes nutritious choices.

**Supplementary Information:**

The online version contains supplementary material available at 10.1007/s13668-025-00660-1.

## Introduction

When mitigating the rising prevalence of diet-related Non-Communicable Diseases (NCD), the environment surrounding individuals plays a significant role in shaping and constraining human behaviour, particularly in food stores where consumers are directly influenced by food displays [1, 2]. It is well-established that consumers are subtly nudged into purchasing certain products, a common marketing tactic to boost sales [1]. Given the constant exposure to this setting, supermarkets naturally serve as a relevant focus for research [3]. Consequently, improving food environments has been proven more effective in reducing socioeconomic inequalities in diets and health compared to individual-focused measures such as nutritional education and healthy eating campaigns [4]. Implementation of supermarket nutrition policies could support the development of healthier food environments for consumers, with a consequently large impact on preventive health strategies [5]. While some supermarkets have begun to introduce such policies [6], a unified approach to address these issues is lacking, with efforts remaining scattered. Therefore, there is an urgent need to establish policies regulating displays in supermarkets.

To begin with, food environments have been extensively studied for their role in influencing the development of NCDs [7]. Among these environments, supermarkets play a pivotal role in shaping dietary choices, particularly in the context of increasing rates of diet-related NCDs [8]. Previous research has explored the complex interplay between the supermarket environment, individual behaviours, and health outcomes [9, 10, 11]. The relationship between supermarkets and consumers’ health is of an undisputable interdependence. Where supermarkets distribute the available options, thereby influencing consumer choices, which in turn shapes purchasing behaviours [12]. As gatekeepers of food environments, supermarkets play a crucial role in determining the accessibility of both healthy and unhealthy food choices. The relationship between supermarkets and consumers plays a crucial role in promoting sustainable food systems. Our research aligns with Sustainable Development Goals (SDGs) 2 and 3, which aim to achieve zero hunger and ensure good health and well-being for all. In line with SDG 1, our goal is to enhance access to healthy and appropriate food while strengthening consumers’ understanding of nutrition. By adopting a systems approach, we seek to address malnutrition by ensuring consumer´s access to healthy food in supermarkets. Furthermore, our focus on SDG 2 underscores the need for a holistic approach that fosters a fairer, more accessible, and nutritious food environment to support consumer well-being.

Consumer awareness of supermarkets’ roles in promoting healthy food environments is evident, with significant support for initiatives aimed at increasing the availability of fresh and nutritious foods [13]. The literature underscores the importance of enhancing food environments within supermarkets to combat the rising prevalence of NCDs [14, 15]. Civil society supports the notion that supermarkets bear responsibility for fostering health-promoting environments through strategic product placement, marketing, and availability [13, 16]. Studies investigating consumer behaviour in retail settings have highlighted the prevalence of marketing strategies aimed at promoting healthy food choices, although significant challenges remain in ensuring consistent adherence to such practices [5, 17]. Seeing the constant and frequent exposure people have to supermarkets, transforming them into healthier environments would have population-scale benefits. Addressing these challenges requires careful consideration of the regulatory landscape. Supermarkets often face obstacles when adapting to new governmental regulations aimed at improving food environments [18]. Acknowledging and navigating these challenges are essential steps in fostering collaborative efforts between policymakers, researchers, and food retailers to promote healthier food environments in supermarkets.

Additionally, effective government policies and actions are crucial for creating healthy food environments that enable healthy diets and reduce obesity, diet-related NCDs, and associated inequalities [19]. Experts emphasize the role of the supermarket environment and consumer behaviour, as well as the assessment of interventions and policies aimed at achieving healthier retail [14]. Therefore, it is important to extend the focus to include the role of private policies in addition to public policies [14]. The literature highlights the need for a harmonized systematic overview and evaluation of policy implementation to create healthier food environments [19, 20]. Simultaneously, we see that supermarkets are increasingly addressing unhealthy food environment requests. Australian supermarkets have corporate social responsibility policies impacting public health nutrition, though many lack specificity and focus primarily on sustainability [21, 22]. Some policies successfully target the reduction or elimination of unhealthy foods at checkouts [23]. Nevertheless, France and Australia have identified a need to improve the Business Impact Assessment of Obesity (BIA-Obesity) as corporate commitments often did not translate into tangible effects on metrics [24, 25].

Finally, public and private policies targeting the food retail sector can contribute to achieving sustainability goals. However, there remains a lack of comprehensive outlining of how supermarket environments can be optimized to promote healthy purchases [26, 27]. Additionally, there is a need to track existing initiatives of companies to increase the adoption of high-potential initiatives and guide future recommendations for supermarket policies. Several articles have presented their benchmarking of nutrition-related policies set by supermarkets, though no unified taxonomy of those has been set in place, leaving a knowledge gap.

The objective of our study is twofold: first, to examine how large supermarket groups incorporate policies to promote healthy and sustainable purchases; and second, to investigate the extent of national policies regulating supermarkets’ initiatives to ensure healthier nutrition environments beyond food safety issues. In this study, we aim to clarify the available policies related to food advertisement, supermarket architecture, and other factors that contribute to creating better food environments. Addressing this knowledge gap is significant as it has the potential to guide future supermarket policy recommendations. One of the main outcomes of this research is providing insights into how ongoing policies can be enhanced and promote reproducibility by competitors to promote the purchase of nutritious and sustainable food items.

## Methodology

We conducted a systematic review to identify governmental and private policies of major supermarket brands in Argentina and Germany, focusing on initiatives to enhance the supermarket environment and promote healthier choices. Though the systematic review was done for both countries, this study appraises and discloses the results from the German sample as a representative country from Europe. The study was preregistered (link omitted for review purposes).

### Sample Choice

This is part of a larger study that focuses on Argentina and Germany, aligning with SDGs 2 and 3, which target zero hunger and good health and well-being, respectively. These countries were selected due to recent national policy shifts, particularly the implementation of new behavioral nutrition policies aimed at improving food choices and diets [28, 29]. However, the results presented will be based on the German sample as a representative sample of Europe, which may differ from the Argentinian data.

There has been growing academic interest in understanding commonalities in supermarket policies, especially regarding sustainable and nutritious consumer choices in Germany. The German Federal Environment Agency emphasizes the need for suitable frameworks to competitively promote sustainable consumption [1]. Their report highlights the importance of raising consumer awareness through in-store interventions in design, product placement, composition, and advertising [1]. Moreover, the recent nutrition strategy in Germany emphasizes improvements in food labelling and packaging, potentially prompting retailers to embrace their responsibility for a more sustainable and nutritious approach [29].

The selection of supermarket groups was based on the market share of the largest supermarkets in each country [30, 31]. For Germany, we selected Rewe, Lidl, Aldi Süd, Aldi Nord, and Edeka, supermarket groups present in most European member states [31].

### Search Strategy and Study Selection

We conducted a systematic review following the PRISMA protocol [32 and developed a comprehensive search strategy to identify relevant studies. Platforms such as Scopus, PubMed, Informas, and the Nourishing Database were utilized. We searched for articles encompassing keywords preestablished which were “supermarket policies”, and “healthy eating” among others, available in the supplementary material 1. With our initial search strategy, we obtained 287 articles. We identified grey literature by searching websites, databases, national government pages, and private supermarket policy documents. Inclusion criteria encompassed studies focusing on public or private policies and strategies implemented by major supermarket brands in the chosen countries, as well as international or national reports and strategies aiming to improve nutrition and food choices. Studies with their publication dated from 2015, release of the post-2015 SDG [33]. Finally, studies published in the English, German or Spanish language were considered.

We did not set any a priori language restrictions, but our search terms were done only in English. After articles written in Spanish or English were processed by our team and the ones in German were translated through document translator tools.

#### Title and Abstract Screening

Upon retrieving articles, we conducted a screening process by applying inclusion and exclusion criteria to titles and abstracts to identify potentially relevant studies. Emphasis was placed on the origin and content of the articles. Only policies and initiatives that had been implemented or completed were included. Due to the study team’s capacity, a single round of abstract screening was conducted. First, we screened the articles based on their content and origin, restricting the search to Germany. The publication date was particularly important when evaluating private supermarket policies [33].

#### Full-Text Screening

Subsequently, we screened studies that aligned with our research questions, assessing their eligibility based on study design and objectives. Full-text articles of selected studies were then retrieved and assessed for final inclusion.

### Synthesis of Findings

Our taxonomy was structured based on the extracted information relevant to policymaking to improve environments. We used the impact modules of the INFORMAS framework as the basis for our taxonomy. We categorized policy initiatives aimed at improving the supermarket environment and promoting healthy choices using the INFORMAS modular structure framework and WHO action areas as guidance [34, 35]. The INFORMAS framework consists of two process modules designed to monitor public and private sector policies and actions, seven impact modules that assess key characteristics of food environments, and three outcome modules that monitor dietary quality, risk factors, and the mortality and morbidity of NCDs.

Using the review manager tool COVIDENCE, we uploaded all initial literature searches retrieved and followed the updated PRISMA model to structure our systematic review [32] (https://www.covidence.org/). With this tool, we developed a layout for our systematic review, including study characteristics, country, study design, specific policies/strategies examined, outcomes assessed/pending, and any relevant findings or conclusions, available in the supplementary material 2. We chose several key items for extraction based on the paper by Van Dam et al. (2022) [25].

#### Analytical Approach

During the information extraction process, we followed our predefined layout, filling the relevant cells accordingly. In some cases, we simply noted the presence or absence of specific characteristics, while in others, we provied a narrative description of the documents. After completing the extraction, we conducted a thorough double-check and approved the data from each document. The finalized dataset was then downloaded as a CVS file provided by COVIDENCE. This approach enabled to compare the extracted data within the COVIDENCE app and systematically organize the variables for quantification. Our final inclusion date was July 2023.

We synthesized the extracted data, summarizing the characteristics and findings of the included studies, focusing on the policies and strategies implemented by major supermarket groups to promote healthy food purchases; and identified common themes, trends, and gaps in the literature. The extraction process prioritized key aspects relevant to current German policy, including product reformulation, labelling schemes, participation of civil society, and food promotion targeted at high-risk populations [24, 24, 28, 34]. We first observed the supermarket policies, extracted the data and dug deeper into the initiatives they mentioned they were doing to improve the consumer´s knowledge of nutrition and have healthier and more sustainable life habits. To do that we did a parallel research of the initiatives presented on the supermarket policies and their webpages (supplementary material 2). Then extracted the information from the other documents to see how these policies were aligned/ regulated/ guided by governmental strategies.

## Results

In this section, we present the developed taxonomy we used to categorize the different sustainable nutrition policy actions we found in the literature. While spotting incentive and regulative public policies, we have focused on and classified the private policies developed by supermarkets. Examining their projects and campaigns designed to improve their food environment and encourage consumers to choose healthy options.

### Overall Characteristics of Included Studies

**Fig. 1:**
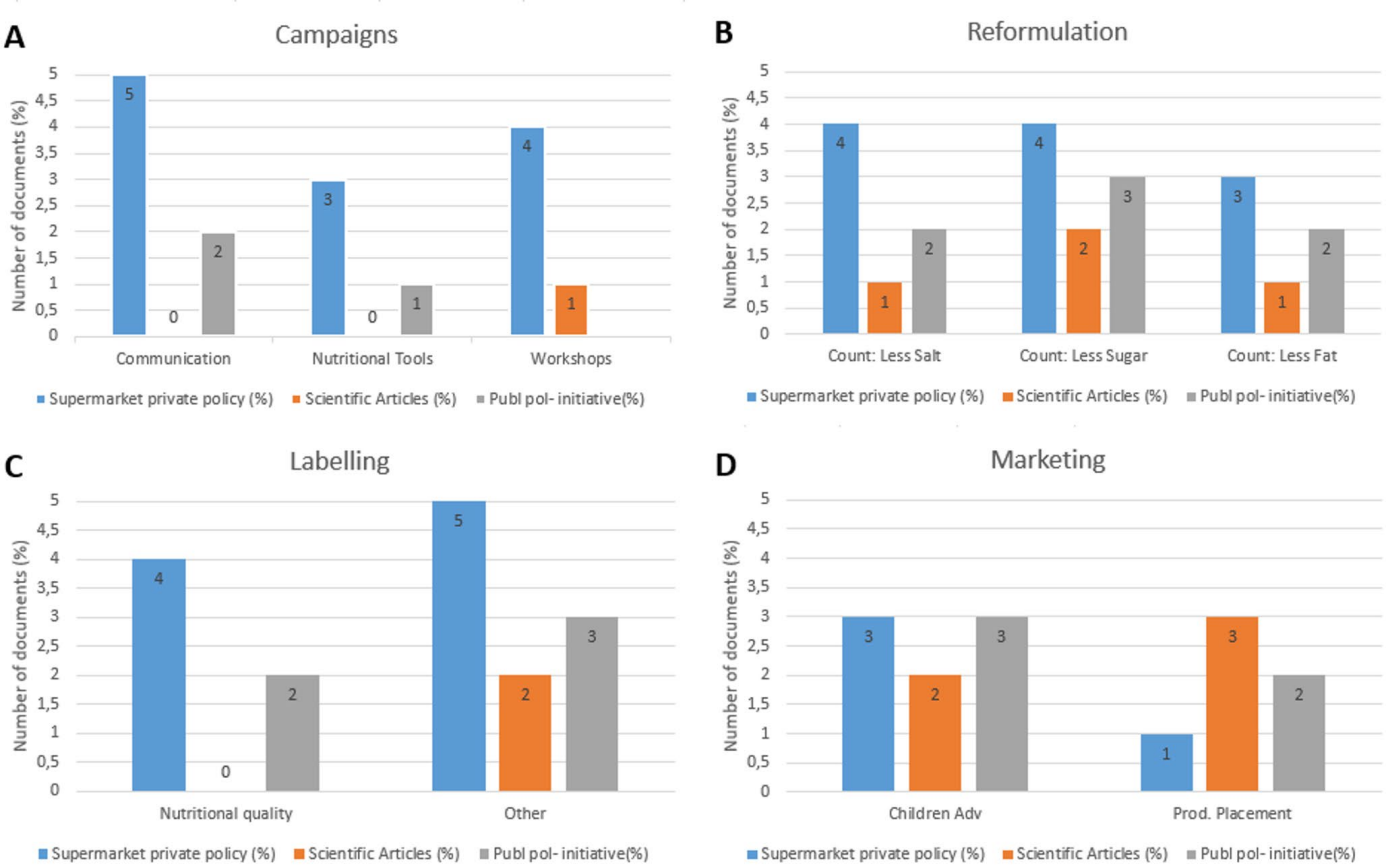
Distribution of subcategories discussed per document group

We extracted the information from 11 documents after the screening analysis of our initial sample. The 45% came from private supermarket policies set by their Supermarket Groups (SG), 27% from scientific documents, and 27% from governmental policies and initiatives fulfilling our eligibility criteria. Figure 1 shows the distribution of our taxonomy´s categories discussed by each document group (private policies, scientific articles and public policies). The categories of food reformulation and marketing were mentioned in all document groups. The results obtained from the governmental policies will be presented under Subquestion.

### Main Research Question

To answer our research question “How and which supermarket groups incorporate policies to promote healthy and sustainable purchases?”, we have extracted the information relevant to policymaking to improve supermarket food environments [25]. Among private supermarket policies, we identified two dedicated specifically to sustainable nutrition (Lidl and Aldi-Sud) and three broader sustainability policies that included specific nutrition chapters (REWE and Aldi-Nord). Edeka, on the other hand, only made general referecneces to healthy nutrition initiatives, as their report primarily focused on the environmental aspects of sustainability.

**Fig. 2:**
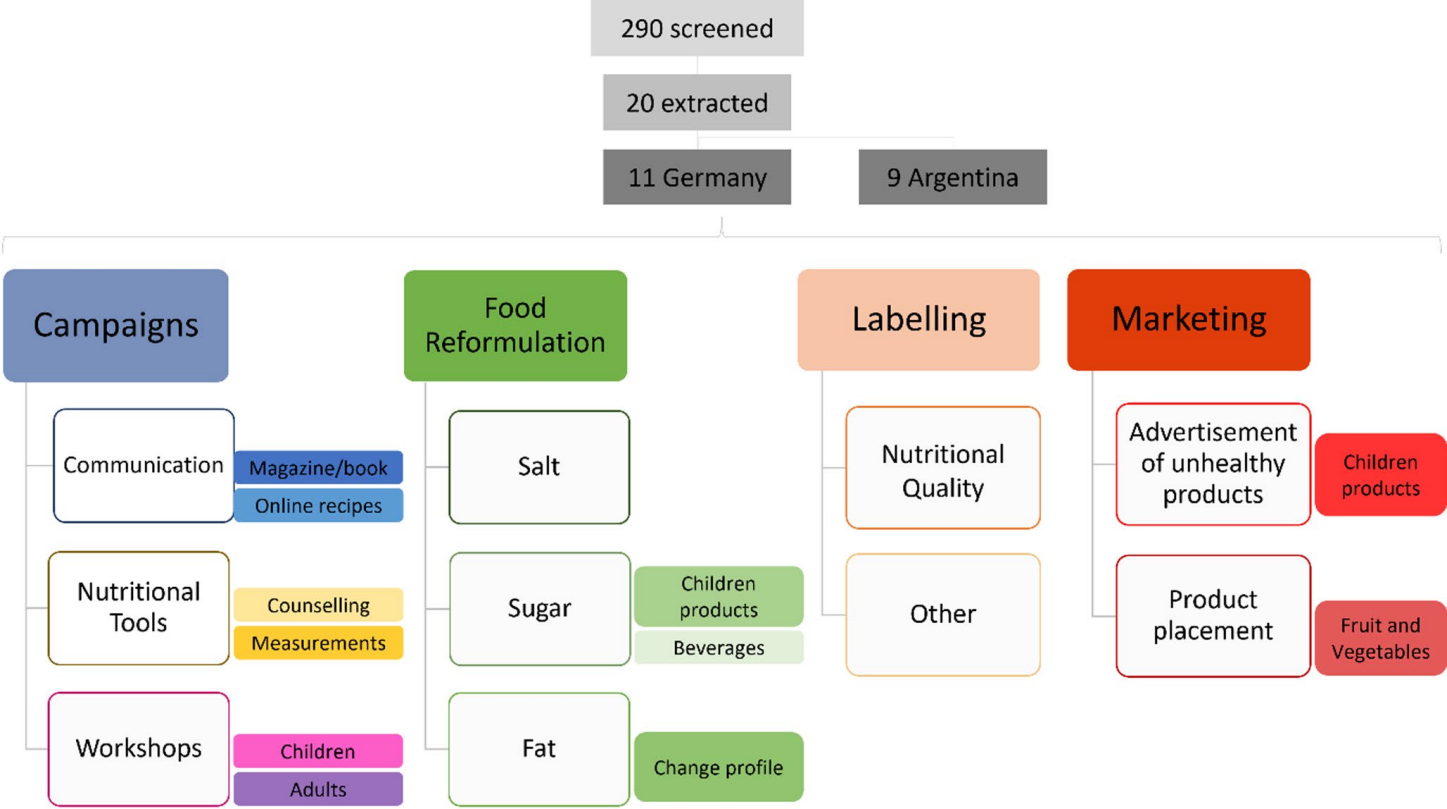
Supermarket policy taxonomy with subcategories for the German sample

Figure 2 presents the final taxonomy, including its various levels or categories. This taxonomy comprised four main categories: campaigns, food reformulation, labelling, and marketing. Subsequently, we divided each category, highlighting key discussion points relevant to sustainable nutrition as outlined in each supermarket policy. Within the campaigns category, the subcategories included communication, nutritional tools, and workshops. Under food reformulation, we identified subcategories focused on the reformulation of nutrients such as salt, sugar, and fat content. For the labeling category, we classified subcategories based on initiatives related to labels indicating nutritional or non-nutritional quality (vegan, organic, biological, animal-welfare labels). Finally, in the marketing category, we included the advertisement of unhealthy products and product placement, key actors relevant to ensure healthier food environments.

#### Campaigns

The various supermarket reports discussed campaigns designed to promote healthier diets among consumers. We conducted an in-depth analysis of these campaigns and performed online searches to identify the topics covered and the approaches used. Access to the sources and distribution of subcategories is provided in the table from the supplementary material 3. It should be noted that no supermarket groups addressed all subsections of communication, nutritional tools, and workshops. Nevertheless, they all explicitly mentioned their communication campaigns, 60% of them developed workshops and only half of them had initiatives where they established nutritional tools to empower consumers’ choices.

##### Communication

We have observed all the supermarket groups underline their communication campaigns using magazines/books to share sustainable nutrition messages. From the analysed documents, Lidl, Edeka and Aldi-Nord specified the use of these communication tools, where topics like nutrition, vegetarianism and veganism are transferred. Specifically, Edeka has a flyer called the “Feel Good Principles” for consumer distribution. In this document, Edeka discusses the importance of a healthier lifestyle where high whole grain foods, lots of fruits and vegetables (F&V), seasonal nutrition, physical activity and mental health tips are introduced and explained (supplementary material 3). Moreover, all the groups provide small linked publications with key information on balanced diets, sustainable nutrition Deutsche Gesellschaft für Ernährung (DGE) tips, and the nutri-score.

Finally, the approach to providing consumers with ideas for cooking was seen as a common currency between all the analyzed supermarket groups. Online recipes are not only a place for inspiration but with the various filters of diet preferences, and dietary restrictions, providing a more straightforward approach that all supermarket groups have implemented.

##### Nutritional Tools

There have been various Nutritional tools presented by the different supermarket groups. There are several initiatives set to give consumers information about their needs and ways to eat healthier, and one has been spotted to give broader information about the food. The first subgroups of this subcategory are related to direct nutritional counselling tools, informing consumers about how to improve their nutrition Fig. 2. The second subcategory presents heath measuring tools that can be of use to learn about their health determinants and help guide how to navigate the food environment.

Several supermarket groups provided consumers with some sort of nutritional guidance. Firstly, we have Edeka´s, REWE´s and Lidl`s “*week-plan*” and “*diet plan*” that give consumers an idea of what would be an ideal dietary plan for their profiles of just some weekly recipe ideas to reduce the cognitive burden of meal planning. Those tactics are complemented with dietary lifestyle habits recommendations in the case of Lidl´s initiatives. Edeka also provides a unique telecentre where consumers can phone and get tailored nutritional advice (supplementary material 3).

Moreover, when it comes to general tools Edeka and REWE have several measurement tools to enable consumers to be more empowered with their own nutrition and health. Edeka and REWE provide consumers with the option of using their BMI calculators. Furthermore, REWE also provides a measuring tool to calculate the nutriscore of recipes. This tool called “ErnährWert” has been developed using the DGExpert and not only presents the nutritional value of products but also entire recipes. It shows a value from 1 to 10 showing how well what you want to cook or bake meets the current Deutsche Gesellschaft für Ernährung (DGE) recommendations, where 1 is the worst adhesion to the recommendations and 10 is the best. Lidl reported that they have something called *“Nutritional overview”*, which is the nutritional summary information of the dishes you get when your diet plan or weekly plan is generated with them.

##### Workshops

Several supermarkets have also created Campaigns that intend to increase the population´s knowledge regarding nutrition topics. We found these campaigns in all the supermarket groups excluding Aldi-Nord. There are several workshops to teach nutritional principles to children, school programs for students and educational resources for teachers. Additionally, the Food Epi analysis by Pinda et al., established that for Germany actions to establish engagement and knowledge transfer between government, civil society, practice, and research are top priorities [20].

First, we have the subcategory of programs aimed at children. REWE created a project called “*Hunger for Goals*”, which teaches children about conscious nutrition and how to grow vegetables while attending a football camp. In collaboration with Tafel, they also provide healthy power boxes for schools to ensure children have a nutritious start. Since 2000, REWE has participated in the 5-a-Day program, integrating its principles into all their workshop initiatives.

Similarly, Edeka has established projects such as “*Vegetable Beds for Kids*” and “*Cooking with Children*,” which offer tips on introducing children to new foods and integrating cooking into daily life. Edeka also developed programs like “*Market Rally”* and “*Shopping Coaching*,” where school classes visit supermarkets to learn about healthier nutrition, and “*Senses Course*” “Health Foods,” and “*Check-dein-Essen*,” which use playful campaigns to introduce children and young people to healthy nutrition. Additionally, Edeka launched the “*Move More - Eat Better*” project, aimed at making 3rd and 4th-grade students curious about nutrition, exercise, and responsibility. This program includes practical content to help adolescents take responsibility for their eating habits, bodies, and health. These initiatives often involve immersion days where students, and sometimes adults, visit supermarkets to learn about products, experience the taste of healthy food, and understand the hidden fat in chips, sugar in energy drinks, and the benefits of whole grain products.

Finally, the subcategory of “Adults” includes the material and workshops created to improve nutrition education in the classroom. The Aldi-Sud has its “*Vegetable Academy*”, where students and teachers receive professional advice to grow vegetables on the field. They tend to the plants and learn about their nutritional qualities. Moreover, Aldi-Sud also has a project “*Campus Ackademie*” where they enable and encourage teachers to incorporate practical knowledge about growing vegetables and nutrition aiming to imprint those topics in the class and nurture students in these areas. The Lidl also has something called “*fruit school*”, which consists of free online nutritional education materials for elementary school teachers and parents. The aim is to show students playfully what a balanced diet with vegetables and fruit looks like and why biodiversity is important for growing them.

#### Product Reformulation

Across the different reports and initiatives, we saw a particular interest in improving food quality from its basal level of recipe composition. Of the five supermarkets analyzed, 80% of them mentioned instances of food reformulation in their products. Only Edeka did not mention their interests or actions in incorporating the reduction of salt, sugar and fat in all their private brands. Some talk about grams of reduction in specific products, some mention that they have “*joined a federal government strategy to reduce sugar*,* fats*,* and salt in ready-to-eat meals*” but are not clear about the actual reformulation change. When we talk about actual reporting of the application of the Supermarket policies, the Food EPI analysis documents mention the governmental introduction of a tax on sugar-sweetened beverages (SSB) based on sugar content and the use of the revenue for health promotion [20]. However, we documented a disparity between policy recommendations and their implementation, particularly in the German context [20]. All the supermarkets that had those reformulation policies advocated for a reduction of salt and sugar, but only 75% of supermarket groups advocated for a reduction in fat content.

##### Sugar

In this category, we included the subcategory of children-oriented candy and foods and sugar reduction for soft beverages.

Specific children-oriented reformulation generally refers to baby food. We want to underline the initiative set by Aldi-Sud who stated that will not back children from sweets but will reformulate them into being “child-friendly“. Moreover, Lidl stated that they will not replace the reduction of Sugar with sweeteners, which seems to be in line with expert nutritional recommendations. They publicly specify that since 2017 they have reduced 15% salt reduction and 13% sugar in their branded products. Finally, this initiative was still being criticised by the paper of Von Philipsborn et al. because of the unclear statements of how that will be achieved and monitored [36].

Sugary beverages are of key importance due to their high sugar content and negligible nutritional value. Only 50% of the supermarkets advocating for product reformulation mentioned intentions to reduce salt and sugar content in the beverages subcategory, including Aldi-Sud and Lidl. These statements were general and lacked specificity regarding quantities or proportions. Specifically, Lidl reiterated that they will not replace sugar reduction with sweeteners.

##### Fat

Generally, when supermarket nutrition initiatives talk about establishing a general Fatty Acid (FA) reduction, they focus on improving the FA profile. Moreover two of them, Aldi-Sud and Lidl, even mention the initiative to improve the profile of FA. Specifically increasing the proportion of unsaturated ones. Nevertheless, they are not clear and precise on how they intend to do it, which products will be affected and what are their objectives.

##### Salt

The salt narrative is repeated along the supermarket policies, stating the need to reduce consumption to care for the consumer’s health and help improve it. We have found out that 80% of our supermarket sample discusses the importance of salt reduction on consumer-oriented products. Where we also saw that Aldi-Sud and Lidl have developed a specific chapter expanding the topic of salt reduction, health implications, and the link between high salt intake with heart disease and hypertension.

#### Labelling

We have divided the labeling group into two subcategories. The first is “Nutrition quality”, labels created to show the products’ positive or negative nutritional composition saliently, and the second is “Others”, where labels included are more environment-oriented. The rationale for not separating this category into more subcategories relies on the main aim of this research. Though we look at Sustainable nutrition Policies, our focus is on healthy products. Therefore, when we talk about labelling that talks about nutritional quality, we can not include the Vegan label as it is not only on healthy vegan products but unhealthy high in FA and Salt products. Only 80% of the groups that had a labelling policy set in place included the incorporation of the Nutritional label as a topic, all of those mentioning the Nutriscore implementation.

##### Nutritional Quality

All the documents searched talk about the importance of having clear nutritional labels. Though the food reformulation strategy stated that the simplified nutrition labelling was going to be implemented by the end of 2019, Food-Epi stated that the application of mandatory labelling in Germany has poor implementation [20]. When it comes specifically to supermarket policies, we have found that all supermarkets, but Edeka, mentioned the implementation of the Nutri-Score in their products and the intention to and their intention to label all their brands by the end of 2023, REWE group, and 2024 Aldi-Nord and Aldi-Sud. Even though Edeka does not mention them in their policy, they talk about them in their communication campaigns to communicate nutritional labelling online. We expect Edeka to follow the competitor’s trend, but as the Nutri-score was not mentioned in their Sustainability report, then we cannot present it here.

##### Others

All supermarket groups mentioned their interest in increasing the incorporation of climate labels and non-nutrition-related ones. The other labels proposed and emphasised by the supermarkets include the vegan logos, animal husbandry, ASC, place of origin and the Bio label of the European Union. All the supermarket groups referred to the Vegan label stating that it would be present in all their privately branded food products. Moreover, they all stated their intention to expand the variety of plant-based products. It is important to underline that the Sustainability report from the Federal Environment Agency that owned brands of supermarkets can be perceived as a label and can increase the complexity of the “*Label Jungle*“ [1]. We present this classification here to clearly distinguish that when supermarkets talk about Sustainable Nutrition Policies they may talk about these labels, and the discourse needs to be clear because many of these labels do not intend to make the consumers´ choice more nutritious or nutritionally aware.

#### Marketing

We found that from the policies supporting the improvement of marketing to help make healthier environments only 75% of the supermarket groups said they were reviewing the advertisement targeting children and 25% mentioned the product placement matter. When we extracted the main data presented in the supermarket documents we saw that the main topics of interest regarding marketing were the Advertisement of unhealthy products to children and the distribution and placement of the food products in general.

##### Children’s Marketing

There is a specific emphasis on the topic concerning Children’s marketing, 62,5% of the documents, excluding governmental initiatives) talked about the importance of regulating advertising to protect children’s nutrition. In general, they specifically state that they will “*Avoid marketing unhealthy products for children and advertise and promote healthy products*”. Where they all state the “*Exception: Promotional items for Christmas*,* Easter and Halloween*”. Yet, only Aldi-Sud and Lidl stated that they were not going to advise any unhealthy products to children that did not fit the WHO recommendation.

##### Product Placement

On the other hand, when it comes to Product placement, 87% of the articles (excluding governmental initiatives) mentioned the importance of caring for product placement and health. The Sustainable Report of German Supermarkets stated that the Country’s supermarkets should focus on influencing individual consumer and shopping behaviour and directing it to more sustainability through “*shop design*” [1]. Moreover, only 20% of the supermarket reports mentioned the need to allow easy access to F&V one of them was the Aldi-Sud and the REWE group. These documents emphasise the importance of large availability and visibility of fruit and vegetable availability and display to favour the transition into Sustainable Diets. They also mention the reincorporation of packaging “*Naturgut*”, “*Pro Planet label*” and green price labels to make high animal welfare and organic products more salient. Finally, the document “*Voluntary Industry Initiatives to Promote Healthy Diets* “ by Peter von Philipsborn expressed how Lidl stated their commitment to reducing portion and package sizes of energy-dense products and products high in sugar and salt [36]. Unfortunately, it is also stated that the pledge does not mention Lidl’s healthy checkout initiative, which was first introduced in its UK stores in 2014. The author proposed that the discrepancy might be due to the lack of attention received among advocacy groups in Germany as compared with the UK.

### Subquestion

Following the creation of the Supermarket nutrition policy taxonomy, we examined the extent of Governmental legislation regulating supermarkets beyond food safety issues. We have analyzed three main documents from the German Ministry of Agriculture and Livestock. Those were the “*Cornerstone paper: Towards a Nutrition Strategy of the Federal Government*”, now we will call it Cornerstone paper; the “*National Reduction and Innovation Strategy for Sugar*,* Fats and Salt in Finished Products*”, now we will call it National reduction strategy; and the “*Promoting sustainability in Food Consumption- Developing an integrated food policy and creating fair food environments*”, now we will call it Promoting Sustainability. [37, 37, 38].

The Cornerstone paper refers to a group of recommendations for the national food and nutrition policy that are coming out in 2024. This paper published in 2022 is an Initiative, guidelines to build a policy strategy. The National Reduction and Innovation Strategy document is the specific policy made to regulate the content of these nutrients in processed products in Germany. The first document was written in 2016 and then several updates were made [39]. We have included the most updated version available for this research. Finally, the Promoting Sustainability document is also a public policy initiative from 2020, that intends to define policies aiming to promote sustainability in food consumption. It is shaped as policies that integrate four targeted dimensions of human health, social aspects, the natural environment and animal welfare.

We understand that the three main governmental documents propose to strongly tackle two of the four categories, reformulation and labelling, and weakly mention and propose to improve the marketing category, specifically when it comes to the advertisement of unhealthy products to children.

This does not mean that there is no mention of campaigns and marketing in these documents but no specific ongoing regulation was found in the extracted documents. It is worth mentioning that we found some mention of the communication campaigns, aiming at the digitalization and clear translation of healthy and sustainable practices from policy and academia to the general population. This was the case for the Cornerstone paper and the Promoting Sustainability papers.

#### Product Reformulation

Upon delving into product reformulation initiatives within public policies and strategies, our research revealed a consensus among all documents advocating the necessity to establish baseline limits for food reformulation for human consumption. The Cornerstone Paper briefly acknowledges the reformulation initiative without delving into further elaboration. In contrast, the National Reduction Strategy and the Promoting Sustainability provided detailed directives for action. Specifically, the National Reduction strategy document sets forth ambitious targets, mandating a double-digit reduction in sugar content within processed products by the year 2025. While it emphasizes the imperative of minimizing fat content to the minimum possible but is not more specific. Pointedly, the policy addressed the issue of reformulation of sweet beverages. The document stressed the importance of follow-up and thorough monitoring regarding the use of sweeteners and other sugar substitutes. Similarly, Promoting Sustainability focuses predominantly on reformulating products high in sugar, with less emphasis on salt and fat reduction strategies. Despite this oversight, it strongly emphasizes the support for the National Strategy of Sugar, Fats and Salt Reduction. They pushed forward the introduction of a tax on sugar-sweetened beverages, the mandatory labelling of beverages with the Nutri score, and the promotion of the consumption of light spritzers through reformulation.

#### Labelling

Parallel to what supermarkets have stated, governments show a high interest in putting clear and easy-to-understand nutritional labels into practice. The three documents state the importance of having a clear labelling system. The Cornerstone paper specifies the significance of the Nutriscore and the implementation of the Label of origin and ecological footprint mentioned. The document even states that to acquire the labels, they have to be based on quality requirements that prevent greenwashing and misinterpretation. It emphasises the Nutritscore as the chosen gold standard for nutritious food signalling. The Reformulation paper stated that: Generally mentions labels and the coalition of the Federal Governments to launch a simplified labelling system by the summer of 2019. It must be noted that they implemented the Nutri-Score by 2020. Promoting Sustainability, again supported Nutriscore and encouraged to introduction of the climate label and animal welfare one.

#### Marketing

In the marketing section, the extracted governmental documents talk about our key subcategories but are not so specific about their regulation of them. We chose two main subcategories in the marketing domain of extreme relevance to support consumers´ healthier food choices, advertisement of unhealthy products and product placement.

Initially, when it comes to advertisements of unhealthy products there is a key interest in the ones targetting children. The Reformulation paper makes various points regarding this matter. It states how the German Central Association of the German Advertising Industry (ZAW) will review and revise its rules of conduct by 2019, focusing on advertising directed at children and social media platforms. Additionally, in responding to EU updates on Audiovisual media service rules, there is a push against advertisements directed at children promoting foods with high sugar, fat, and salt. Moreover, the Policy recommendation clearly states, in recommendation 6, that they strongly advocate for implementing measures to regulate such marketing strategies. Finally, there is a slight mention of the matter in the Cornerstone paper which primarily focuses on the further development of the National Reduction and Innovation Strategy for sugar, salts, and fats in ready-made products.

When it comes to product placement the nutrition initiative, as outlined in the Cornerstone paper, stated the need to promote and create appropriately beneficial nutrition environments that facilitate healthy eating patterns across various life stages and settings * – “from infant and child feeding to the company canteen and the supermarket shelf”.* Emphasising the promotion of plant-based diets rich in unprocessed vegetables, fruits, high-fiber grain products, legumes, and nuts. Additionally, the Promoting Sustainability document acknowledges the significance of product placement in shaping food environments, specifically mentioning the transition needed where *“animal products are primarily replaced with more vegetables and legumes”.* But also proclaims that while governmental actions influence food environments, particularly through information and labelling policies, other factors such as product placement, retail outlet locations, and pricing policies are predominantly influenced by companies along the food chain. The Reformulation paper did not touch upon this topic.

## Discussion

Effective government policies and actions are key to creating healthy food environments that enable healthy diets and reduce the pervasive levels of obesity, and diet-related NCD [19]. Nevertheless, we have seen several supermarkets that have developed their private policies and initiatives to ensure that certain SDGs are being met. In this systematic review, we dug deep into those documents and governmental policies that intend to stimulate sustainable nutrition practices to improve our population’s health.

Our study focus was to develop and design our taxonomy intending to understand and identify which supermarket policies were tackling key topics related to the improvement of food settings in supermarkets. We discovered that the main categories of campaigns and labelling were developed in all the analysed supermarket groups’ policies, whereas food reformulation and marketing categories were in 80%. Our analysis underscores the widespread adoption of sustainable nutrition policies by most supermarket groups, signifying a collective acknowledgement of the importance of fostering healthier dietary choices. However, we note a notable exception in the case of the Edeka group, highlighting variations in indirect commitment across different stakeholders within the industry. Despite this, the development of private sustainable nutrition policies across supermarket chains demonstrates a shared commitment to promoting health-conscious consumer behaviours. These results are in line with research found in other countries such as Australia and France where there is an underlying interest from these large supermarket groups to participate in the general transformation of more sustainable and nutritious stores [24, 25 The paper published by Sacks et al. pointed out the importance of identifying and encouraging supermarkets to work towards developing healthier environments [24, 40]. Their findings agreed with our stating that four large supermarket groups have set policies related to obesity and improving nutrition, and are demonstrating leadership in doing so [24, 40].

### Campaigns

All supermarket groups engaged in communication campaigns, primarily through recipes, magazines, and flyers, aiming to educate consumers about balanced diets and sustainable nutrition principles. Consumers seek helpful nutrition information such as recipe ideas for easy-to-prepare healthy meals, which supermarkets provide uniformly [41].

However, there was variability in the provision of more interactive tools and workshops. It was particularly interesting to find that several supermarkets offered some form of eating recommendation service, providing guidance to both consumers and employees. Additionally, after our analysis had concluded, Lidl introduced a new tool called “NährwertKompass”, which follows REWE’s example of a nutritional tool designed to estimate the healthiness of recipes [42]. While some supermarket groups provided nutritional counselling services, diet plans, and health measurement tools, others lacked such personalized resources. However, there appears to be a ripple effect, with the introduction of such tools.

Workshops targeting children were more common than those for adults, reflecting a focus on early intervention and habit formation. These initiatives go beyond the simple assurance of safe and nutritious environments in the supermarket. It almost seems to be some kind of change in the supermarkets’ mission from mere food providers to supporters of the SDG and consumers’ well-being. Nevertheless, we understand that the interest may also rely on the companies finding this caring for social impact indicators as a business opportunity in response to investor behaviour [43].

### Product Reformulation

Food reformulation is a complex process for supermarket groups, as their retail work is linked to various companies and industries where they have little control over the end product´s composition. Nevertheless, the proportion of own-branded products in the studied supermarket groups is larger than half, with canned goods at 64% and dairy products at 54% ^1^. Here we see a great opportunity for supermarket groups to tail those products, reformulate them according to national health requirements and, improve the salience and availability of those healthier products.

We would like to highlight the opportunity to capitalize on the common approach that supermarket groups seem to have, particularly given the flexibility in product reformulation, which can make products healthier, more sustainable, and more nutritious. In a study by Martinez et al., supermarkets were found to have a high level of autonomy when making decisions about food retail strategies. The study involved individuals responsible for in-store decisions about retail practices who participated in online surveys and semi-structured interviews. Overall, retailers expressed a willingness to engage in healthy food retail and a desire for greater support from healthy food retail initiatives [21].

Unlike similar research, we found that all supermarket group policies in our German sample discussed reformulation following the Nutri score guidance [25]. Notably, 80% of our sample mentioned their alignment with the reformulation policy and official nutrient profiling recommendations. Unfortunately, despite governmental initiatives such as the introduction of a sugar tax on sugar-sweetened beverages based on sugar content in Germany, the low implementation level of these policies poses a significant challenge to realizing the intended health promotion benefits [20]. That must change as several studies stated the benefit of those policies, due to the possible aversion of type 2 diabetes and other NCDs when including taxation schemes on SSB [44, 45, 46].

### Labelling

Though we are conscious of the relevance of knowing how animal welfare and vegan products are represented in supermarkets our urgent worry, due to the overweight and obesity epidemic, is on human welfare rather than animal welfare and vegan labels.

Both, supermarket groups and governmental policies, strongly supported the implementation of clear nutritional labelling systems, with the Nutri-Score emerging as the preferred standard. The incorporation of this system is of key importance because though Nutri-Score labelling is not compulsory yet, the initiative of the supermarket groups is that they have already established it in the majority of their own branded products. The introduction of health and nutritious labelling has been shown to drive the reformulation of products to make them healthier [47, 48].

We did not intend to analyse the other labels due to the focus on the nutritional size of the matter. We see though, that there was interest in introducing labels for origin, ecological footprint, climate impact, and animal welfare, making us concerned about the potential greenwashing and misinterpretation effect all the labels together may have on our consumers [1, 49].

### Marketing

From an unhealthy promotion of products to children we eagerly observe the initiative made by 80% of the supermarket group’s policies. Nevertheless, we also see that just a minority of the papers talk about following the WHO recommendation for marketing products to children and therefore critically observe the need to improve the reporting and measuring. The consequences of unrealistic and scattered policies can lead to inefficient implementation and lack of effect [20]. Similar papers agree that the *“Profuct and Brand Promotion*” need to strengthen their commitment and application of initiatives to limit marketing to children [25]. We saw that the complete sample of our governmental documents talked about the intention to regulate and set a policy that limits the advertisement of unhealthy products to children. According to Dillman Carpentier et al., this topic should not only be discussed but a clear policy should be urgently implemented [50]. A successful example of this policy is the case of South Corea regarding cartoon advertising to children, where the legislation has led to a decline in the exposure of children to food-related marketing [51]. Though the regulation has not been published and was not available at the time of this research´s development, we know that the German government is currently drawing up legislation to restrict the marketing of unhealthy food items to kids which may be included in their new nutritional policy [52].

Moreover, our analysis identifies a gap in addressing the importance of the physical layout of supermarkets in promoting sustainable nutrition. The role of supermarkets influencing consumer behaviour through store design and product placement remains underemphasized in existing policies. Positive effects on healthier food choices have been found when leveraging infrastructure mechanisms, supermarkets can enhance the visibility and accessibility of nutritious food items, thereby steering consumer choices towards healthier options [17]. The absence of specification in the sustainable nutrition supermarket policies when it comes to the physical layout of supermarkets and product placement is not original from us. Australian companies presented limited commitment towards increasing healthy *“Product accessibility” by the company groups* [24]. Growing evidence shows the significant effect this can have on the increase of sales of Healthy products and the decrease of confectionary for example [53]. Another example is the one set by Ejlerskov et al., which focused on feasibility and the need to create policies that focus on the reduction/ elimination of unhealthy foods at checkouts [23]. While there is insufficient evidence base relating to the role of the foodscape in terms of the obesity crisis; policy and civic society and industry must work together and take action now, in areas where current evidence suggests change is required [54].

### Takehome Message from Governmental Influence

The Farm to Fork Strategy [55], and the WHO European Food and Nutrition Action Plan 2015–2020 [56] agree that more ambitious food environment policies are required for countries to achieve global nutrition targets. In Germany, governmental nutrition strategies have primarily focused on measures such as reformulation and labelling. However, these represent some of the policies with the lowest implementation of a healthy food environment in Europe [20, 59]. Nevertheless, the development of additional well-structured policies could create a successful framework to make the dietary changes necessary for healthier food environments. For instance, a harmonised policy could support supermarkets’ initiatives by reinforcing consistency.

Although regulating supermarket initiatives through public policy offers significant opportunities, supermarkets face additional challenges which can difficult that process. Addressing these challenges requires careful consideration of the regulatory landscape of each country. For example, in Australia, researchers have seen that public policy initiatives targeting supermarkets often face difficulties with implementation and maintenance [21, 22]. One of the main barriers is the concern among both small and large retailers that compliance with new regulations could threaten financial viability and customer satisfaction, particularly if certain products no longer meet policy standards and are consequently removed from shelves [18, 60]. However, the proposed approach in this study does not seek to remove products from the market, but rather to oversee and coordinate existing initiatives implemented by retail groups. Nonetheless, misunderstanding or lack of awareness of the policy objectives may still present significant obstacles for retailers [18, 60].

In the German context, the implementation of existing public retail initiatives remains limited. For example, zoning laws for unhealthy food outlets (e.g., fast food restaurants), zoning laws for healthy outlets (e.g., greengrocers, farmers’ markets), support for healthy in-store environments, and policies promoting healthier food service settings all show low uptake levels [59]. Despite this fragmented policy landscape, these initiatives share a common goal: to improve food environments. This presents an opportunity to support retail actors from a logistical perspective, thereby helping to advance these national objectives.

Acknowledging and navigating these challenges are crucial steps in fostering collaborative efforts between policymakers, researchers, and food retailers to promote healthier food environments in supermarkets. To improve policy comprehension, governments could adopt a consultancy-based approach in which retailers receive implementation support as part of the policy itself [18, 60]. For example, a nutrition expert could be fixed within retail teams to assess the healthiness of procured food products [18]. Additionally, financial incentives could encourage necessary adjustments while promoting equity among retailers of different sizes. Involving retailers in feasibility testing may further enhance private sector engagement and foster long-term collaboration [60]. Moreover, public policies could complement private initiatives by setting monitoring standards, enabling further transparency [61].

In Germany, the last recent policy change is the “Lieferkettensorgfaltsgesetz”, which focuses on social dimensions along the entire value chain and the EU Sustainability Reporting Directive starting in 2024 to regulate the reporting on sustainability indicators. Based on the systematic review findings, we have reported on the policies already developed by supermarket groups to make their environments more nutritious.

In summary, our study sheds light on the integration of sustainable nutrition policies by supermarket groups and the role of governmental legislation in shaping healthier, more sustainable nutrition environments. While supermarkets have demonstrated a commitment to promoting healthy and sustainable purchases, there is a need for greater coherence and alignment in monitoring and reporting mechanisms to follow up on their achievements and compare them between supermarket groups. Additionally, governmental strategies could benefit from this already established common goal of developing a comprehensive approach that addresses all categories of supermarket environments, to ensure more support in promoting healthier food choices. A proposed research area would be the behavioural policy approach exploring the role of frictions or hurdles in the supermarket food environment [62, 63]. Such frictions, called sludges, can severely hinder healthy food choices.

Future research could explore how both large and small supermarket groups can modify their food environments to promote healthier choices, implementing affordable and practical changes suited to their respective capacities. Additionally, supplementary studies could examine the feasibility of implementing healthy retail nutrition policies through collaboration between supermarket groups and policymakers.

### Limitations

We recognise the limitations of a systematic review, particularly the challenges associated with relying on self-reported, open-access private policies. This reliance may introduce bias and inaccuracies, as reports could overstate achievements or initiatives. Additionally, while our study focuses on research domains related exclusively to the German population, we acknowledge that the selected supermarket groups operate at a European level, making our developed taxonomy applicable beyond Germany. Furthermore, our study did not include small-scale supermarkets, which may face greater challenges in implementing changes to create healthier environments due to limited resources compared to larger supermarket groups. However, the study applies to most supermarkets, as German food retail is dominated by 4 companies that share 90% of the market [31].

Looking ahead, future research should focus on evaluating the effectiveness of these initiatives, identifying best practices, and addressing gaps in implementation and enforcement. Additionally, we foresee the need for an in-depth policy analysis, tracking each policy’s objectives and accomplishments over time.

## Conclusion

This paper analyses how supermarkets are improving the quality of their service keeping in mind and tackling unhealthy food environments to some extent. Our taxonomy is comprised of four main categories: campaigns, reformulation, labelling, and marketing. Subsequently, we segmented each category, delineating key discussion points relevant to sustainable nutrition as outlined in each supermarket policy. For the first one, the campaigns category, the subcategories included where communication, nutritional tools and workshops. Then the second category was subdivided into salt, sugar, and fat reformulation subcategories. Continuously, the third one was split into the nutritional or non-nutritional quality labels; followed by the marketing category, which included the advertisement of unhealthy products and product placement.

Our study provides insights into supermarket policies aimed at promoting healthier food environments that can be used as a leverage point to the development of actionable, unified future supermarket policies. Ensuring that the recommendations address the scientific need to tackle obesogenic food environments while considering and including the work and experience already set by supermarket groups and their policies. As we care for animal welfare and nurture characteristics, we see an urgent need to focus on human welfare, where the purchase environment is healthy and promotes nutritious choices.

Under these voluntary policies set by supermarkets, policymakers should consider the initiatives and ensure a more organized transition, taking into account the retailers’ trade-offs and opportunities. Without such coherence, it becomes challenging to gauge the success of these initiatives and identify areas for improvement. Furthermore, showing how the government seems to take a part in those privately set policies, opening the question about the possibility of supermarket group policies and governmental participation, which could be provided by doing a unified reporting strategy. We foresee the opportunity to foster collaborative efforts between policymakers, researchers, and food retailers to promote healthier food environments in supermarkets.

## Key References List

The references are presented with their number fitting to the reference list on the manuscript. The justification for the choice is written in grey colour, underlined and in italics.

Importance:

1. Federal Environment Agency. *How Sustainable Are German Supermarkets*. https://www.umweltbundesamt.de/sites/default/files/medien/479/publikationen/texte_107-2022_wie_nachhaltig_sind_die_deutschen_supermaerkte.pdf (2022).

*This was the spark of the initial paper design. After studying the report*,* the gap for assessing the role of Supermarkets in providing better food environments was clear. The public health relevance and importance of the matter were shaped on the paper.*

11. Shaw, S. C., Ntani, G., Baird, J. & Vogel, C. A. A systematic review of the influences of food store product placement on dietary-related outcomes. *Nutr. Rev.* nuaa024 (2020) doi:10.1093/nutrit/nuaa024.

*Shows how the change of the architecture impacts the food choice*,* consequently consumer health*.

19. Caputo, V. & Just, D. R. The economics of food-related policies: Considering public health and malnutrition. in *Handbook of Agricultural Economics* vol. 6 5117–5200 (Elsevier, 2022).

*It underlines the opportunity and importance of behavioural policy to address a broad range of public health issues. Specifically presenting the available literature regarding various interventions designed to improve the population´s nutritional health*,* including voluntary reformulation of food store´s environment.*

49. BMEL. “Good Food for Germany” - the Federal Government’s Food and Nutrition Strategy. https://www.bmel.de/EN/topics/food-and-nutrition/food-nutrition-strategy.html#:~:text=On%2017%20January%202024%2C%20the%20Cabinet%20adopted%20the%20Federal%20Government's, commenced%20on%2010%20November%202023. (2024).

*With the development of the new Strategy*,* our research´s relevance is presented with the opportunity to support the German government´s aims.*

51. Lake, A. A., Moore, H. J., Cotton, M. & O’Malley, C. L. Opportunities to improve population health: possibilities for healthier food environments. *Proc. Nutr. Soc.***82**, 264–271 (2023).

*A broad picture of the background of unhealthy food environments. In addition to a clear definition of the different actors*,* initiatives*,* and hot discussion points around healthy food environments. Stating how the last ones are needed to improve our population´s health. The paper also goes through our taxonomy´s categories.*

55. European Commission. *Europe’s Beating Cancer Plan*. https://health.ec.europa.eu/system/files/2022-02/eu_cancer-plan_en_0.pdf (2022).

*It underlines the urgency to improve the environments and touches upon all our Taxonomy categories and most of our subcategories.*

Outstanding Importance:

7. Vadiveloo, M. K., Sotos-Prieto, M., Parker, H. W., Yao, Q. & Thorndike, A. N. Contributions of Food Environments to Dietary Quality and Cardiovascular Disease Risk. *Curr. Atheroscler. Rep.***23**, 14 (2021).

*The accentuation regarding the importance of modifying food environments and promoting healthier food choices*.

18. Law, K. K., Pulker, C. E., Healy, J. D. & Pollard, C. M. “Just So You Know, It Has Been Hard”: Food Retailers’ Perspectives of Implementing a Food and Nutrition Policy in Public Healthcare Settings. *Nutrients***13**, 2053 (2021).

*Underlines Supermarkets’ struggle to fulfil healthy nutrition standards*,* promoting healthier habits. It outlines the problem’s complexity but also sheds some light on the transformation process we could see in the sector.*

24. Sacks, G. et al. Benchmarking the Nutrition-Related Policies and Commitments of Major Food Companies in Australia, 2018. *Int. J. Environ. Res. Public. Health***17**, 6118 (2020).

*References 24 and 25 both took the presence of nutrition policies in supermarkets in France and Australia and critically analyzed the initiatives. They complement perfectly our developed taxonomy*,* and they are what I hope for a follow-up on this paper.*

25. Van Dam, I. & Vandevijvere, S. Benchmarking the nutrition-related commitments and practices of major French food companies. *BMC PUBLIC Health***22**, (2022).

*References 24 and 25 both took the presence of nutrition policies in supermarkets in France and Australia and critically analyzed the initiatives. They complement perfectly our developed taxonomy*,* and they are what I hope for a follow-up on this paper.*

50. Federal Environment Agency. *How Sustainable Are German Supermarkets*. https://www.umweltbundesamt.de/sites/default/files/medien/479/publikationen/texte_107-2022_wie_nachhaltig_sind_die_deutschen_supermaerkte.pdf (2022.)

*Due to the chosen experimental approach of this pilot study*,* where our research question is somehow represented*,* it was shown how the display change(Having more fruits and vegetables and on a strategic position) improved the nutritious household purchase and dietary quality.*

## Electronic Supplementary Material

Below is the link to the electronic supplementary material.

Supplementary Material 1

Supplementary Material 2

Supplementary Material 3

## Data Availability

No datasets were generated or analysed during the current study.
