# Supplementary material for: Beyond Food Safety: Taxonomization of Private Initiatives to Design Healthier Supermarket Environments
Source: Curr Nutr Rep. 2025 May 28;14(1):71. doi: 10.1007/s13668-025-00660-1 (PMC12119755; doi:10.1007/s13668-025-00660-1)
Supplement: Supplementary file 1 — Supplementary Material 1 [file 13668_2025_660_MOESM1_ESM.docx]

# Supplementary material 2. Data extraction layout template.

**General information**

**Title of paper/abstract / report that data are extracted from:**

**Lead author contact details:**

**The country in which the study conducted**

1. Germany
2. Argentina

**Initiatives**

**Labels**

1. Octagonal Warning labels
2. Nutriscore
3. Organic Labels
4. Own Brands
5. All Brands
6. Other: *Space to write*

**Data of policy application extent and results:** *Space to write*

**Product Reformulation**

1. Less Salt
2. Less Sugar
3. Less Fat
4. Other: *Space to write*

**Unhealthy product marketing restrictions/advertising bans**

1. Yes
2. No
3. Other: *Space to write*

**Product Placement**

1. Healthy checkouts
2. Easy access Fruit and vegetables
3. Other: *Space to write*

**Share of Nutritious/sustainable Products (Meat, F&V, Vegan, Legumes, Whole Grain):** *Space to write*

**Sweet beverages representation:** *Space to write*

**Initiatives to increase nutrition awareness set by supermarkets** *(In store/ online/out-of-store)*

|  | **Name** | **Description** |
| --- | --- | --- |
| **Initiative 1** |  |  |
| **Initiative 2** |  |  |
| **Initiative 3** |  |  |
| **Initiative 4** |  |  |
| **Initiative 5** |  |  |
| **Initiative 6** |  |  |
| **Initiative 7** |  |  |
| **Initiative 8** |  |  |
| **Initiative 9** |  |  |

**Characteristics of included studies**

**Methods**

**Aim of study:** *Space to write*

**Design**

1. Qualitative study
2. Public Policy
3. Private Policy
4. Initiative
5. Case report
6. Other: *Space to write*

**Start date:** *Space to write*

**Possible conflicts of interest for study authors:** *Space to write*

**Population description:** *Space to write*

**Inclusion criteria:** *Space to write*

**Exclusion criteria:** *Space to write*

**Total number of documents:** *Space to write*
