# Supplementary material for: Beyond Food Safety: Taxonomization of Private Initiatives to Design Healthier Supermarket Environments
Source: Curr Nutr Rep. 2025 May 28;14(1):71. doi: 10.1007/s13668-025-00660-1 (PMC12119755; doi:10.1007/s13668-025-00660-1)
Supplement: Supplementary file 2 — Supplementary Material 2 [file 13668_2025_660_MOESM2_ESM.docx]

# Supplementary Material 1: PRISMA Supermarket Policies

Keywords: "supermarket" "grocery store" "food retailer” "Initiative" "policy" "strategy" "sustainable nutrition" "healthy eating"

**Identification**

References from other sources **(n =39)**

Grey literature (web pages supermarkets/government page)

Studies screened **(n = 208)**

Studies sought for retrieval **(n = 69)**

Studies assessed for eligibility **(n = 69)**

References removed **(n = 79)**

Duplicates identified manually (n =7 )

Duplicates identified by Covidence (n = 72 )

Studies excluded **(n = 139)**

Studies not retrieved **(n = 0)**

Studies excluded **(n = 49)**

Wrong outcomes (n = 8)

Wrong Population (n = 34)

Wrong intervention (n = 1)

Wrong study design (n = 7)

**Included**

Studies included in review **(n = 20)**

**Screening**

References from databases/registers **(n = 248)**
