# Supplementary material for: Beyond Food Safety: Taxonomization of Private Initiatives to Design Healthier Supermarket Environments
Source: Curr Nutr Rep. 2025 May 28;14(1):71. doi: 10.1007/s13668-025-00660-1 (PMC12119755; doi:10.1007/s13668-025-00660-1)
Supplement: Supplementary file 3 — Supplementary Material 3 [file 13668_2025_660_MOESM3_ESM.docx]

# Supplementary material 3: Supermarket group Campaigns and their subcategories

| **Subcategory** | **Communication** | | | **Nutritional Tools** | | | | | **Workshops** | | |
| --- | --- | --- | --- | --- | --- | --- | --- | --- | --- | --- | --- |
| **Key topics** | **Online Recipies** | **Books/Magazines/Flyers** | | **Nutritional Counselling** | | | **Health measuring tools** | | **Children** | | **Adults** |
| **EDEKA group** | Recipes ^1^ | VollVegan ^2^ | Feel good principles ^3^ | Weekly plan ^4^ | | | BMI calculators ^5^ | Recommended caloric intake ^6^ | Vegetable beds for kids ^2^ | Market rally, Shopping coaching, Check-your-food ^7^ | |
| **Lidl group** | Recipes ^8^ | Lidl-cooking Magazine^9^ | | Weekly plan^10^ | Diet plan ^11^ | | Nutritional overview ^12,13^ | | Fruit school^14^ | | |
| **Aldi Nord group** | Recipes ^15^ | Conscious Nutrition ^16^ | | Absent | | | | | | | |
| **Aldi Süd Group** | Recipes ^17^ | Conscious Nutrition ^18^ | | Absent | | | | | Vegetable Academy^19,20^ | | Campus Academy^20^ |
| **REWE Group** | Recipes ^21^ | Your kitchen ^22^ | Knowledge about Nutrition ^23^ | Weekly plan ^24^ | Dietary recommendations ^25^ | Diet plan^26^ | BMI calculators ^27^ | ErnährWert ^28^ | Workshops 5 a day ^28^ | | Absent |

## Sources

1. EDEKA. Online Rcipies. *Rezepte* https://www.edeka.de/rezepte/suche.jsp (2023).

2. EDEKA. *EDEKA GROUP Anual Report*. https://verbund.edeka.de (2022).

3. EDEKA. Feel good principles. https://www.edeka.de/static/media/S%C3%BCdwest/Regionale-Medien/Homepage/Ern%C3%A4hrungsservice/Ernaehrungsservice_Broschuere_2021_Web.pdf (2023).

4. EDEKA. Wochenplan. *Meal-Prep-Wochenplan: Vorlage zum Vorkochen, Rezepte und Tipps* https://www.edeka.de/ernaehrung/lebensmittelwissen/superfoods-trends/meal-prep-wochenplan.jsp (2023).

5. EDEKA. BMI calculators. *BMI-Rechner: bestimmen Sie Ihren Body-Mass-Index* https://www.edeka.de/ernaehrung/tests-und-rechner/bmi-koerpergewicht-pruefen.jsp (2023).

6. EDEKA. Recommended caloric intake. *Kalorienverbrauch berechnen – Wie viele Kalorien verbrennen Sie beim Sport?* https://www.edeka.de/ernaehrung/tests-und-rechner/kalorienverbrauch-berechnen/index.jsp (2023).

7. EDEKA. Nutritional service. *EDEKA-Ernährungsservice* https://www.edeka.de/suedwest/ernaehrungsservice.jsp (2023).

8. Lidl. Online Rcipies. *Rezepte* https://www.lidl-kochen.de/ (2023).

9. Lidl. Lidl-Kochen Magazin. https://www.lidl-kochen.de/kochideen/ (2023).

10. Lidl. Wochenplan. https://plan.lidl-kochen.de/#/plan/06-12-2023 (2023).

11. Lidl. Dietplan. *Dein Essensplan – Einkaufen und Kochen leicht gemacht mit dem Lidl Wochenplaner* https://www.lidl-kochen.de/ernaehrungsplan-erstellen/ (2023).

12. Lidl. Nutritional overview. https://www.lidl-kochen.de/ernaehrungsplan-erstellen/abnehmen/ (2023).

13. Lidl. *Lidl Einkaufspolitik*. https://unternehmen.lidl.de/verantwortung/positionspapiere (2022).

14. Lidl. Fruit school. *Fruchtschule* https://unternehmen.lidl.de/pressreleases/2021/210616_fruchtschule (2021).

15. Aldi Nord. Online Rcipies. *Rezepte* https://www.aldi-nord.de/rezepte.html (2023).

16. Aldi Nord. Conscious Nutrition (Food pyramid, Vitamins and minerals role, kids nutrition). https://www.aldi-nord.de/ratgeber-tipps/bewusste-ernaehrung.html (2023).

17. Aldi Süd. Online Rcipies. *Rezepte* https://www.aldi-sued.de/de/rezepte.html ) (2023).

18. Aldi Süd. Conscious Nutrition (How to eat consciously, Contious nutrition to children). https://www.aldi-sued.de/de/nachhaltigkeit/bewusste-ernaehrung.html (2023).

19. Aldi Süd. Vegetable Academy. https://www.aldi-sued.de/de/nachhaltigkeit/soziales-engagement/gemueseackerdemie.html (2023).

20. Aldi Süd. *ALDI_Sud_Nutrition_Report_2023_EN*. (2023).

21. REWE. Online Rcipies. *Rezepte* https://www.rewe.de/rezepte/ (2023).

22. REWE. Deine Küche. https://eu-assets.contentstack.com/v3/assets/blt085f7ef3068b29af/blt07d3a0c53443d02a/65d35951dff895040a80f3a5/Internet_0224.pdf (2023).

23. REWE. Wissen mit Nährwert. https://www.rewe.de/ernaehrung/wissen-lexikon/ (2023).

24. REWE. Weekly food plan. *Der perfekte Diätplan* https://www.rewe.de/ernaehrung/wochenplan-essen/ (2023).

25. REWE. Dietary recommendations to Lose weight. *Abnehmen* https://www.rewe.de/ernaehrung/abnehmen/ (2023).

26. REWE. Nutritional plan. *Kostenlose Ernährungspläne* https://www.rewe.de/ernaehrung/ernaehrungsplan/ (2023).

27. REWE. BMI calculator. *BMI-Rechner* https://www.rewe.de/ernaehrung/bmi-rechner/ (2023).

28. REWE. *REWE Group Sustainability Report*. www.rewe-group-nachhaltigkeitsbericht.de/2022 (2022).
